# Arthroscopic Treatment of Stiff Elbow

**DOI:** 10.5402/2011/378135

**Published:** 2011-07-06

**Authors:** Davide Blonna, Enrico Bellato, Eleonora Marini, Michele Scelsi, Filippo Castoldi

**Affiliations:** Umberto I-Mauriziano Hospital, University of Turin Medical School, Largo Turati 62, 10128 Torino, Italy

## Abstract

Contracture of the elbow represents a disabling condition that can impair a person's quality of life. Regardless of the event that causes an elbow contracture, the conservative or surgical treatment is usually considered technically difficult and associated with complications. When the conservative treatment fails to restore an acceptable range of motion in the elbow, open techniques have been shown to be successful options. More recently the use of arthroscopy has become more popular for several reasons. These reasons include better visualization of intra-articular structures, less tissue trauma from open incisions, and potentially the ability to begin early postoperative motion. The purpose of this paper is to review the indications, complications, and results of arthroscopic management of a stiff elbow.

## 1. Introduction

The main functions of the elbow are to position the hand in space and to act as a stabilizer for actions such as carrying, throwing, pushing, pulling, and lifting. In order to accomplish its function the elbow needs a full or almost full range of motion (ROM). The normal arc of motion of the elbow is from 0 to 145 degrees of flexion [1]. In a biomechanical study Morrey [2] concluded that an arc of motion between 30 and 130 degrees is enough to achieve 90% of the activities of daily living excluding sports and work activities. Consequently, a stiff elbow has been defined as an elbow with a reduction in extension greater than 30 degrees and/or a flexion less than 120–130 degrees [3].

Stiffness of the elbow is not a rare event and it can frequently lead to significant functional impairment which can be challenging to treat. This makes prevention mandatory. However, if prevention fails, a nonoperative treatment, such as physiotherapy or splitting, is usually recommended as the first therapeutic approach. After at least 6 months of such an unsuccessful conservative treatment, surgery may be indicated [4]. 

For many years open capsular release had been the standard treatment for elbow contractures [5]. More recently the use of arthroscopy has become more popular for several reasons including better visualization of intra-articular structures, less tissue trauma from open incisions, and potentially the ability to begin early postoperative motion [5–7].

The worldwide use of arthroscopic techniques has resulted in reports of intraoperative nerve injuries. These severe complications have led some authors to raise serious concerns about the safety of this procedure [5].

The aim of this paper is to review the etiologies, indications, complications, and results of arthroscopic management of a stiff elbow. This comprehensive approach was undertaken to permit a better understanding of the risks and benefits of choosing between open and arthroscopic techniques for stiff elbow.

## 2. Etiologies and Classification

**Figure 1:**
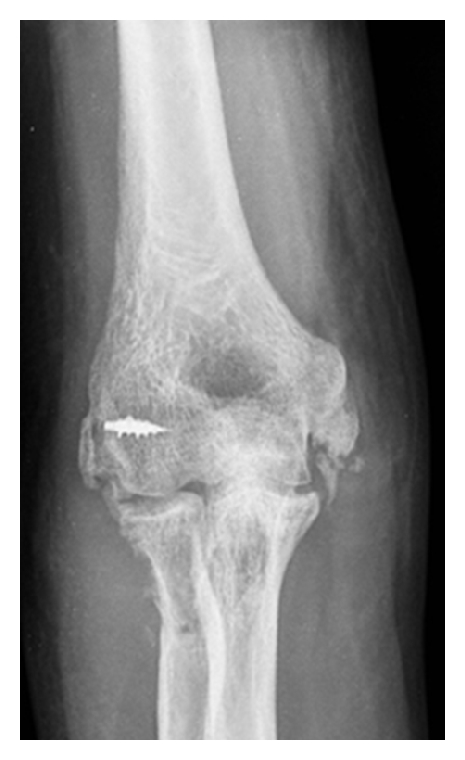
The figure shows an X-ray of a right elbow, 50 days after an LCL repair. The ROM was 80° in extension and 110° in flexion.

Classification and treatment of a stiff elbow are generally based on etiology [4]. Elbow stiffness may be due to either traumatic or atraumatic events. Atraumatic causes of stiff elbow include iatrogenic stiffness (Figure 1), rheumatoid arthritis, osteoarthritis, postseptic arthritis, hemarthroses in hemophiliacs, pterygium syndrome, and congenital contractures, such as in arthrogryposis, and congenital radial head dislocation [8].

It has been suggested that trauma can lead to elbow stiffness both directly and indirectly [8]. Primary traumatic processes include articular surface damage, intra-articular fractures, osteochondral defects, or loose bodies. These processes can cause contracture by themselves or in association with secondary events like capsule, ligaments, and muscles contracture. All these events can eventually lead to elbow osteoarthritis.

Posttraumatic osteoarthritis of the elbow is in fact a common cause of elbow stiffness. An injury to the elbow's articular surface can result in its degeneration with a secondary formation of osteophytes which can hinder elbow motion. Simultaneously bleeding, edema, granulation tissue formation, and fibrosis can cause a thickening of the capsule as well as the elbow's collateral ligaments or a loss of soft tissue compliance [9]. 

Regardless of the events that cause the stiffness, one distinction with important clinical and therapeutic consequences is whether the cause was intrinsic or extrinsic. Common intrinsic (intra-articular) causes include post-traumatic arthritis, joint incongruity, ankylosis of articular surfaces, articular adhesions, loose bodies, and osteoarthritis. Common extrinsic (extra-articular) causes include heterotopic ossification (HO), capsular contracture of scar, collateral ligament contracture, and musculotendinous contracture (most commonly the triceps) [10].

The classification proposed by Morrey is based on this principle [11]. It classifies elbow stiffness as extrinsic and intrinsic and mixed referring to those extrinsic contractures resulting from intrinsic causes.

**Figure 2:**
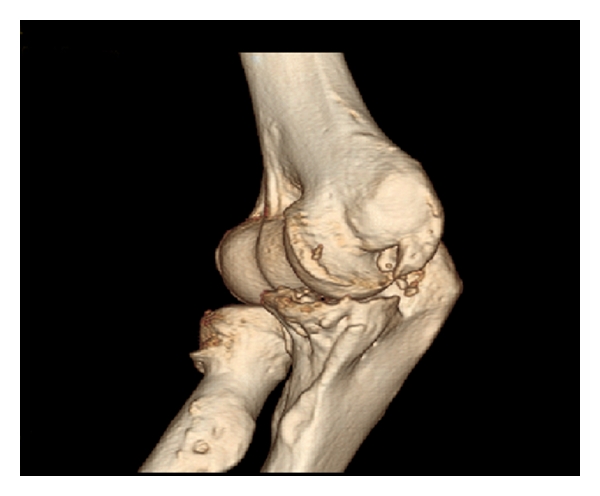
The figure shows the 3D reconstruction of the elbow of a 35-years-old patient with a heterotopic ossification of the medial collateral ligament. The ROM was 45° in extension and 110° in flexion.

Heterotopic ossification (HO) (Figure 2) is a common cause of elbow stiffness with a severe and usually negative impact on possible patient outcomes. It consists of a formation of mature lamellar bone within nonosseous tissues [10]. There are many factors involved in the genesis of HO including elbow trauma, head injuries, burns, fibrodysplasia ossificans, progressive, and iatrogenic conditions [12–15]. 

Trauma is, however, the most common cause of HO. The incidence of post-traumatic heterotopic ossification ranges from 1.6% to 56%, depending on the severity of injury [12]. The incidence has been reported to be five times greater (20%) in cases of fracture dislocation [16]. If the trauma is associated with a head injury, the incidence increased to 76–89%, while in cases of an isolated head injury it is 5–10% [15, 17, 18].

## 3. Indications and Contraindications

Many authors have described arthroscopic techniques to treat a stiff elbow with the aim of regaining a normal motion of 30° to 130° [19]. Based principally on the data published by Morrey et al. [2], surgical release of contractures has traditionally been indicated only for patients with an extension of less than 30 or 35 degrees and a flexion of less than 130° [4, 19, 20].

This indication is not however applicable to all patients. Although most people can in fact lead normal lives with a functional arc of motion of the elbow, young and highly demanding patients (usually athletes) cannot tolerate even small degree of contraction. For these patients an arthroscopic treatment can be indicated to treat even less severe contractures [21].

### 3.1. Contraindications

Elbow arthroscopy is a technically demanding procedure with a long learning curve. Therefore a surgeon's limited experience is generally considered a relative contraindication. 

Another relative contraindication is represented by an altered neurovascular anatomy (e.g., previous ulnar nerve transposition or an extra-articular deformity that may damage vessels or nerves). However a recent study [22] has shown that a previous ulnar nerve transposition or ulnar nerve subluxation does not preclude an arthroscopic treatment. Finally, arthroscopy is not the primary choice to treat an isolated loss of forearm rotation [23].

### 3.2. Technique

Several techniques have been described for the arthroscopic treatment of a stiff elbow. We have used the technique proposed by O'Driscoll [1]. Professor O'Driscoll has taught us that the entire procedure is based on few principles.

- Use a standard and reproducible technique.
- Perform a prophylactic ulnar nerve decompression to avoid delayed onset ulnar neuropathy.
- Constantly control the fluid inflow to avoid swelling.
- Remove the bone in order to recreate conforming joint surfaces.
- Remove the capsule.
- Use retractors.
- Stay under your learning curve.

The patient is positioned in the lateral decubitus position with the shoulder and elbow flexed to 90°. 

Three standard portals are generally used posteriorly: posterolateral, posterior, and direct midlateral (“soft spot”). An accessory proximal posterolateral portal is generally used for retraction. 

Three portals are routinely used anteriorly: anterolateral and proximal anteromedial portals for the working instruments and scope, respectively, and the proximal anterolateral portal for a retractor. Occasionally a second retractor is used and it is placed in the anteromedial portal.

**Figure 3:**
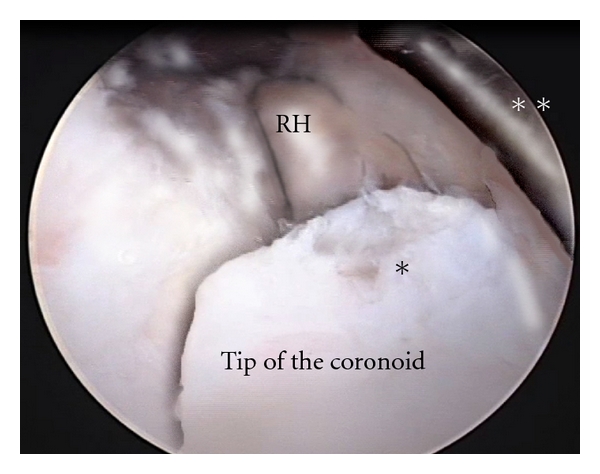
Intraoperative picture of the anterior part of the elbow joint. Heterotopic ossifications are visible on the tip of the coronoid, limiting flexion. The camera is placed in the proximal anteromedial portal. A retractor is placed in the anteromedial portal. *Heterotopic ossification. **Retractor. RH: radial head.

In order to gain flexion a removal of the anterior osteophyte or HO is mandatory (Figure 3). For the most severe contractions the posteromedial capsule can be released through a small incision over the cubital tunnel which permits concurrent ulnar nerve decompression. 

**Figure 4:**
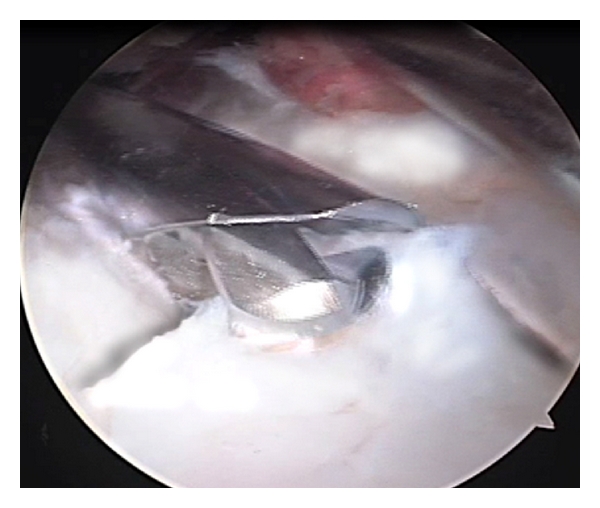
The heterotopic ossification is removed using a 4.5 mm burr.

**Figure 5:**
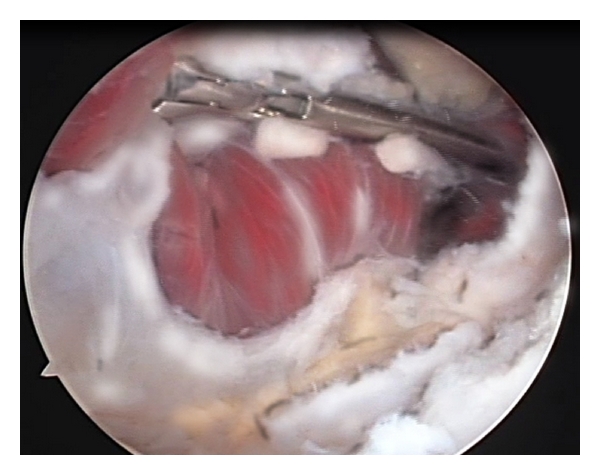
Capsulectomy is performed from medial to lateral with a basket. The camera is placed in the anterolateral portal while the duck-billed basket punch in the anteromedial portal. A retractor is placed in the proximal anterolateral portal.

For a lack of extension both an anterior capsulectomy and removal of the osteophyte in the olecranon fossa is mandatory (Figures 4 and 5).

### 3.3. Postoperative Treatment

No clear information is available in the literature regarding the most effective postoperative treatment for arthroscopic elbow contracture release. Based on what we have learned from Professor Shawn O'Driscoll, we suggest the use of continuous passive motion (CPM) that should be started as soon as possible and continued for at least three weeks.

## 4. Results and Complications

A computer-assisted search was performed using the MEDLINE (from 1985 to 2010) databases to search for the most meaningful data of results and complications related to arthroscopic capsular procedures (capsular release, capsulectomy, and capsulotomy).

### 4.1. Results

The literature on arthroscopic release of the elbow lacks randomized controlled trials. The available studies consist mainly of retrospective studies on small and heterogeneous populations. Despite that, short- and mid-term outcomes in post-traumatic and degenerative arthritis are encouraging. Several reports in fact have documented the efficacy of arthroscopic release of elbow contractures.

Kim et al. [24] analyzed 25 patients affected by loss of motion caused by post-traumatic or degenerative arthritis, with a mean followup of 25 months (12–46). The mean arc of motion gain was of 24°. The mean Mayo Elbow Performance Index (MEPI) [29] improved from 2.8 to 4.6 after surgery, and 92% of the patients were satisfied with the procedure.

Similar good results were reported by Phillips and Strasburger [25]. The authors analyzed 25 patients with elbow contracture caused by post-traumatic arthritis in 15 cases and degenerative arthritis in 10 cases, with a mean followup of 18 months (6–34). The authors reported that the post-traumatic group achieved better results with a mean gain of 50°, while the degenerative arthritis group had a mean gain of only 27°. 

More recently, Ball et al. [27] reported a retrospective series of 14 patients all affected by post-traumatic elbow contracture. The minimum follow up was of 1 year (12–29 months). The mean arc of motion gain was of 41.5°. At the last followup the average pain level measured on a VAS was 3.25, the average self-reported satisfaction score measured on a VAS was 8.4. The ASES functional ability score for the elbow improved in all patients, with an average score of 28.3 (25–30) out of 30 at the latest followup. The authors suggest that this technique has a minor effect on elbow stability when compared to open surgery and obtains better results in flexion contractures rather than in extension contractures. No major complications were reported, except for a case of superficial portal-site infection which recovered completely with drainage and antibiotics.

Kelly et al. [28] reported a series of 25 patients affected by a loss of motion caused by primary osteoarthritis in 21 cases, rheumatoid arthritis in 1 case, and post-traumatic arthritis in 3 cases. The mean follow up was of 67 months (24–123). The mean arc of motion gain was of 21°. The average pain level measured on a VAS scale decreased from 7 to 2 postoperatively. Using the objective/subjective rating scale of Andrews and Carson [30], 14 elbows were scored as poor, 10 as fair, and 1 as good before the operation; while 14 elbows were scored as excellent, 7 as good, 3 as fair, and 1 as poor at the last followup. The overall gain was 37 points in the subjective part of the score and 24 points in the objective part. The authors suggest that this is due to pain relief after the removal of impinging osteophytes both anteriorly and posteriorly. No major complications or second interventions were reported. 

Table 1 summarizes the outcomes of arthroscopic treatment of a stiff elbow.

### 4.2. Complications

Arthroscopy is being used with increasing frequency to diagnose and treat elbow pathologies; the number of elbow arthroscopies has more than doubled in the past decade and now comprises 11% of all arthroscopic procedures [33].

Many anatomical studies highlight the risks of elbow arthroscopy, due to the extreme closeness of the portals with vascular and nerve structures surrounding the joint [41–43]. 

A review of the literature shows an overall complication rate of 6% to 15% with approximately half of those being neurological [33, 44]. 

Although elbow arthroscopy is a relatively safe procedure, the reported complication rate (10%) is higher than that seen with knee and shoulder arthroscopy (1% to 2%) [35].

The reported complications for elbow arthroscopy include compartment syndrome, septic arthritis, superficial infection, persistent drainage from portal sites, and, most frequently, nerve injuries (transient or permanent) [33]. 

Kelly et al. [33] classified complications as (1) those occurring during the surgical procedure and identifiable immediately postoperatively (nerve injury, compartment syndrome, haematoma, or instrument breakage), and (2) those which develop over time (loss of motion, persistent drainage, or superficial infection at a portal site or joint infection). 

Major complications include permanent nerve injury, compartment syndrome, postoperative joint infection, vascular injury, and a loss of motion greater than 30°. 

One of the most common minor complications is transient neurapraxia, with the ulnar nerve being the most susceptible. Nerve palsies are more frequently associated with the execution of capsular release and the case of rheumatoid arthritis [45].

Kelly and colleagues [33] published a series on such complications following 473 elbow arthroscopies and found that there were only four major complications (0.8%) and 50 minor complications (11%). All four major complications were joint space infections, and the minor complications varied from persistent drainage to transient nerve palsy.

In the literature, other reports of complications consist of case studies or brief descriptions of relatively small series of patients. 

Generally nerve injuries after arthroscopic release are rare. Kim et al. [24] reported two transient median nerve palsies in a patient with an elbow contracture on whom an arthroscopic capsular release was performed. Jones and Savoie [31] reported a posterior interosseus nerve transection in a patient with elbow contracture who underwent arthroscopic capsular release. Haapaniemi et al. [32] reported a case of complete transection of median and radial nerves in a patient with post-traumatic elbow contracture treated by arthroscopic capsular release. Nguyen et al. [6] reported a medial antebrachial cutaneous neuroma in a patient with elbow contracture who underwent arthroscopic capsular release.

Park et al. [34] reported a transient thermal injury of the radial nerve, due to an electrocautery device, in a patient with degenerative elbow contracture which was treated by arthroscopic anterior capsular release. Gay et al. [35] reported an ulnar nerve transection in a patient who underwent a revision arthroscopic contracture release.

The surgeon's experience and familiarity with these arthroscopic techniques are perhaps the most important factors in preventing neurovascular complications during arthroscopic debridement. Moreover the use of retractors is likely one of the most important recent advances in preventing nerve injury [1]. 

Comparing complication rates between open and arthroscopic capsular release is difficult since there are no direct comparative studies in the literature [5]. Historically, the literature suggests a low complication rate following open elbow capsulotomy but this data does not seem to be confirmed after a more careful reading (Tables 2 and 3). 

After reviewing the more meaningful articles available in the literature we found that the complication rate among the techniques is comparable. The arthroscopic procedures seem however to be associated with a higher percentage of permanent injury than open techniques. 

Three transient nerve palsies (2 radial, 1 ulnar) were observed in 15 patients following an open, anterior capsulotomy. All were resolved over a course of 3 weeks to 7 months. No infections were noted [36]. 

Husband and Hastings [37] noted transient paresthesias of the ulnar nerve in 1 of 7 patients following an open capsulotomy through a lateral approach that resolved themselves spontaneously.

Marti et al. [38] noted transient ulnar paresthesia in 7 patients after progressive surgical release of a posttraumatic stiff elbow, none of which caused disability in daily living and which disappeared during rehabilitation. Four patients suffered from recurrent stiffness.

Tan et al. [39] illustrated complications after open release for elbow contracture and in particular they highlighted: wound infection (*n* = 3/52 = 6%), cubital tunnel syndrome (*n* = 3/52 = 6%), and reflex sympathetic dystrophy (*n* = 1/52 = 2%). 

Katolik et al. [40] reported 2 cases of anterior interosseous nerve palsy after open release for elbow contracture. In both cases weakness of flexor pollicis longus (FPL) and flexor digitorum profundus (FDP) spontaneously disappeared after approximately 7 months.

## 5. Conclusion

The arthroscopic treatment of a stiff elbow is safe and effective when performed by surgeons with an appropriate level of surgical skills. Compared to open techniques such treatment allows for better visualization and treatment of intra-articular causes of the contracture. The complication rates between the two techniques seem to be comparable. However permanent neurological complications have been reported more frequently with the arthroscopic technique. Further studies are needed to address whether the complication rates of the arthroscopic technique are justified by better clinical outcomes.

## Figures and Tables

**Table 1:** Review of the most meaningful outcomes in term of range of motion related to arthroscopic capsular procedures (capsular release, capsulectomy, and capsulotomy).

		Preoperatory			Follow-up months	Postoperatory		
		Extension (deg)	Flexion (deg)	Arc (deg)		Extension (deg)	Flexion (deg)	Arc (deg)
Kim et al. [24]		21	113	92	25 (12–46)	14	130	116
Phillips and Strasburger [25]		31,5	118,2	87,2	18 (6–34)	6,8	134,6	128,2
Savoie et al. [26]		40	90	50	32 (24–60)	8	139	131
Ball et al. [27]		35,4	117,5	82,1	(12–29)*	9,3	133	123,6
Nguyen et al. [6]		38	122	84	25 (12–47)	19	141	122
Kelly et al. [28]		20	131	111	67 (24–123)	9	141	132

*Average not available.

**Table 2:** Review of the most meaningful neurological complications related to arthroscopic capsular procedures (capsular release, capsulectomy, capsulotomy).

Authors	Nerve injured	Details	Complication rate	Recovery
Jones and Savoie [31]	Radial nerve	Transection	8% (1/12)	Permanent
Haapaniemi et al. [32]	Median and radial nerves	Transection	Case report	Permanent
Kelly et al. [33]	Ulnar, radial, medial antebrachial cutaneous nerve	Nerve injury	16.4% (12/73)	Complete recovery within 6 months
Nguyen et al. [6]	Medial antebrachial cutaneous nerve	Neuroma after transection	4.5% (1/22)	Permanent
Park et al. [34]	Radial nerve	Thermal injury by electrocautery device	Case report	Complete within 12 months
Gay et al. [35]	Ulnar nerve	Transection	Case report	Permanent

**Table 3:** Review of the most meaningful neurological complications related to open capsular procedures (capsular release, capsulectomy, capsulotomy).

Authors	Nerve injured	Complication rate	Recovery
Urbaniak et al. [36]	Radial and ulnar nerve	20% (3/15)	Complete within 7 months
Husband and Hastings [37]	Ulnar nerve	14% (1/7)	Complete
Marti et al. [38]	Ulnar nerve	15% (7/46)	Complete
Tan et al. [39]	Ulnar nerve	6% (3/52)	Complete
Katolik and Cohen [40]	Median nerve	Case report	Complete within 7 months
